# Aberrantly Expressed Embryonic Protein NODAL Alters Breast Cancer Cell Susceptibility to γδ T Cell Cytotoxicity

**DOI:** 10.3389/fimmu.2020.01287

**Published:** 2020-06-19

**Authors:** Gabrielle M. Siegers, Indrani Dutta, Eun Young Kang, Jing Huang, Martin Köbel, Lynne-Marie Postovit

**Affiliations:** ^1^Department of Oncology, University of Alberta, Edmonton, AB, Canada; ^2^Department of Pathology and Laboratory Medicine, Foothills Medical Centre, University of Calgary, Calgary, AB, Canada; ^3^Department of Biomedical and Molecular Sciences, Queen's University, Kingston, ON, Canada

**Keywords:** gamma delta T cells, gammadelta, NODAL, triple negative breast cancer, invasive ductal carcinoma, MICA, tumor evasion

## Abstract

Gamma delta (γδ) T cells kill transformed cells, and increased circulating γδ T cells levels correlate with improved outcome in cancer patients; however, their function within the breast tumor microenvironment (TME) remains controversial. As tumors progress, they begin to express stem-cell associated proteins, concomitant with the emergence of therapy resistant metastatic disease. For example, invasive breast cancers often secrete the embryonic morphogen, NODAL. NODAL has been shown to promote angiogenesis, therapy resistance and metastasis in breast cancers. However, to date, little is known about how this secreted protein may interact with cells in the TME. Herein we explore how NODAL in the TME may influence γδ T cell function. We have assessed the proximity of γδ T cells to NODAL in a cohort of triple negative breast tumors. In all cases in which γδ T cells could be identified in these tumors, γδ T cells were found in close proximity to NODAL-expressing tumor cells. Migration of γδ and αβ T cells was similar toward MDA-MB-231 cells in which NODAL had been knocked down (shN) and MDA-MB-231 scrambled control cells (shC). Furthermore, Vδ1 γδ T cells did not migrate preferentially toward conditioned medium from these cell lines. While 24-h exposure to NODAL did not impact CD69, PD-1, or T cell antigen receptor (TCR) expression on γδ T cells, long term exposure resulted in decreased Vδ2 TCR expression. Maturation of γδ T cells was not significantly influenced by NODAL stimulation. While neither short- nor long-term NODAL stimulation impacted the ability of γδ T cells to kill MCF-7 breast cancer cells, the absence of NODAL resulted in greater sensitivity of targets to γδ T cell cytotoxicity, while overexpression of NODAL conferred resistance. This appeared to be at least in part due to an inverse correlation between NODAL and surface MICA/B expression on breast cancer target lines. As such, it appears that NODAL may play a role in strategies employed by breast cancer cells to evade γδ T cell targeting, and this should be considered in the development of safe and effective γδ T cell immunotherapies.

## Introduction

Breast cancer is the most common women's cancer in Canada with 27 400 expected diagnoses and a projected mortality rate of 6.1% of all cancer deaths in 2020 (1). Mortality is often due to treatment resistance, leading to recurrence and metastatic spread (2).

Immunotherapy using conventional or chimeric antigen receptor-transduced (CAR) T cells is on the cutting edge of advancement in cancer therapeutics (3, 4). However, γδ T cell immunotherapy constitutes an exciting alternative, offering several advantages over the use of conventional αβ T cells. Most importantly γδ T cells are broadly reactive to cancer cells but are not typically MHC-restricted, and thus do not cause graft-vs. host disease (5, 6). Their potent anti-cancer activity and excellent safety profile (7), combined with their non-reliance on tumor mutational loads (8), and improved expansion protocols (9–13) are catapulting γδ T cells into the limelight (14, 15).

Gamma delta T cells kill a wide range of malignancies (6). Their role in breast cancer has been recently reviewed (16). Specific to breast cancer, expanded γδ T cells kill MDA-MB-231, MCF-7 and T47D breast cancer cell lines (17–22). In a phase I clinical trial testing γδ T cell agonist Zoledronate in combination with IL-2 in advanced metastatic breast cancer patients, a significant positive correlation between peripheral γδ T cell numbers and clinical outcome was observed (23). Migration of infused γδ T cells to breast cancer tumors and metastases has been evidenced in both xenograft models (24) and patients (25).

While γδ T cell frequency in blood correlates with positive outcome (23), their prognostic value in breast tumors is unclear. In a comprehensive study including over 18,000 human tumors across 25 cancers, γδ T cell tumor infiltrating lymphocytes (TIL) were the most significant positive prognostic factor (26), although it has since been shown that the CIBERSORT algorithm used in this analysis could not properly discriminate γδ T cells from CD4+ and CD8+ T cells or NK cells; an optimized deconvolution that can reliably identify Vγ9Vδ2 TIL has now been reported (8). The authors of this study focussed on acute myeloid and chronic lymphocytic leukemias, colorectal and prostate cancers, confirming that γδ T cell TIL associate with positive patient outcome, but they did not reassess outcomes for breast cancer patients, which would be of great interest here (8). While a 2012 study proposed that γδ T cells are negative prognosticators in human breast cancer (27), a more recent investigation of TIL in breast cancer using various unbiased *in silico* approaches found that higher levels of γδ T cells correlated with better outcomes (28). In all cases, correlations were identified, but causality not determined.

Later studies have delved more deeply into the presence of γδ T cells infiltrating triple negative breast cancers (TNBC), revealing increased presence of γδ T cells compared to fibroadenomas or breast tissues from healthy individuals, suggesting active infiltration of γδ T cells into tumors (29), and that infiltrating γδ T cells are likely active (30).

The seemingly paradoxical data on γδ T cells in breast cancer highlight the importance of determining the role of γδ T cell TIL before γδ T cells are further developed as a cellular immunotherapy for breast cancer. Indeed, researchers now recognize the importance of determining how the TME influences the function of γδ T cells [reviewed in (31)]. We recently investigated γδ T cell function under hypoxia, a biophysical condition present in many tumors, and discovered that while γδ T cells were activated under low oxygen, breast tumor cells shed MICA to evade detection by γδ T cells (22).

NODAL is an embryonic morphogen secreted by tumor cells in the TME, whose aberrant expression is induced under hypoxia (32). NODAL has been correlated with breast cancer progression, and functionally promotes angiogenesis, invasion, tumor growth and metastasis, irrespective of ER, PR or HER2 status (33–36). NODAL promotes tumor growth in Nude mice bearing a partial immune system, but this effect diminishes when more immunodeficient models are used (33), suggesting a role for NODAL in immune evasion.

Thus, we decided to investigate whether γδ T cells can be found in proximity to NODAL expressing breast tumor cells in TNBC cases and, if so, what impact NODAL may have on γδ T cell function.

## Materials and Methods

### Ethics Statement

This study was carried out in accordance with the recommendations of the Research Ethics Guidelines, Health Research Ethics Board of Alberta—Cancer Committee with written informed consent from all subjects. All subjects gave written informed consent in accordance with the Declaration of Helsinki. The protocol was approved by the Health Research Ethics Board of Alberta—Cancer Committee.

### Patients and Tissues

We assessed 20 surgically resected triple negative breast tumors from cancer patients diagnosed at the Cross Cancer Institute, Edmonton, AB in 2017. Patient and tumor characteristics are listed in Table 1.

**Table 1:** Characteristics of triple negative breast cancer cohort.

	***n***	***n* (% of 9 cases**
	**(% of 20 cases)**	**with γδ TIL)**
**Age at diagnosis—Median (range)**	67.5 (48–91)	63.6 (50–91)
**Histology**		
Invasive ductal carcinoma (IDC)	14 (70)	5 (56)
Multifocal IDC	5 (25)	3 (33)
Apocrine carcinoma	1 (5)	1 (11)
**IDC size (cm)—Median (range)**	1.7 (0.6–5.5)	1.1 (0.6–5.5)
Not specified	7 (35)	2 (22)
<2	7 (35)	4 (44)
2–5	5 (25)	2 (22)
>5	1 (5)	1 (11)
**Tumor grade**		
Not specified	1 (5)	
2/3	2 (10)	1 (11)
3/3	17 (85)	8 (89)
**Tumor stage**		
Not specified	2 (10)	
1	7 (35)	4 (44)
2	7 (35)	3 (33)
3	2 (10)	1 (11)
4	2 (10)	1 (11)
**Lymph node status**		
Positive	9 (45)	3 (33)
Negative	11 (55)	6 (67)
**Deceased as of February 2020**	4 (20)	1 (11)

### Immunohistochemistry

We performed anti-human T cell antigen receptor (TCR)δ staining as reported (22, 37); however, we modified the protocol such as to perform dual staining for TCRδ and CAIX using the EnVision G12 Doublestain System, rabbit/mouse (Agilent Technologies Canada, Mississauga, ON, Canada). Briefly, 4 μm serial sections from formalin-fixed paraffin-embedded tumors were melted at 60°C for a minimum of 10 min on a slide warmer followed by de-paraffinization using fresh Citrus Clearing Solvent (Richard Allan Scientific Reagents, Kalamazoo, MI, USA). Hydration of sections was achieved with a series of graded ethanol (100, 95, 70, 60%) followed by brief incubation in water, then tris-buffered saline plus 0.05% Tween-20 (TBST). Target retrieval solution pH 9 (DAKO North America, Carpinteria, CA, USA) was utilized for antigen retrieval at 100°C for 20 min. After cooling to room temperature, tissues were circled with an ImmEdge pen (Vector Laboratories, Burlingame, CA, USA) and blocking and staining steps were performed as per the manufacturer's instructions. Primary antibody dilutions were 1:150 mouse monoclonal anti-human TCRδ antibody (clone H-41, Santa Cruz Biotechnology, Dallas, TX, USA) and 1:50 dilution of rabbit monoclonal anti-human CAIX [clone EPR4151(2), abcam, Cambridge, MA, USA] or corresponding isotype control diluted to the same antibody concentration. We included known positive controls and isotype controls with each batch for quality control. DAB chromogen bound anti-mouse HRP to indicate TCRδ-positive cells in brown; CAIX-positive cells were stained with permanent red chromogen. After staining with primary and secondary antibodies, we counterstained with Haematoxylin (DAKO), slides were rinsed in water and then dehydrated using a graded ethanol series (60, 70, 95, 100%). Slides were then cleared with Citrus Clearing Solvent, dried and coverslips mounted with VectaMount permanent mounting medium (Vector Laboratories). Serial sections were stained for NODAL as previously published (33).

### Assessment of γδ T Cell Infiltration and Localization With Respect to NODAL and CAIX

**Figure 1:**
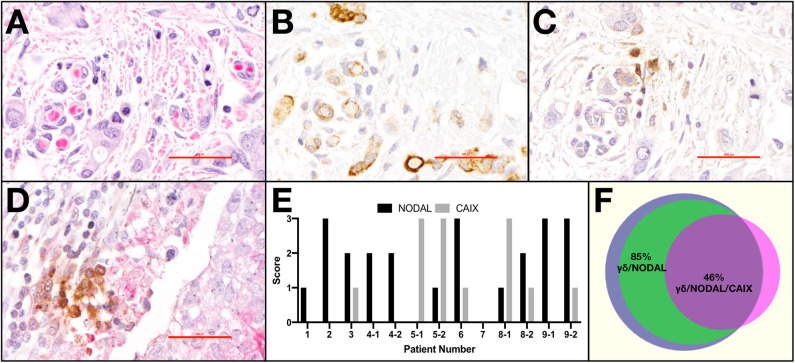
γδ T cells and NODAL are co-localized in breast tumor tissues from patients. Representative example of paraffin-embedded serial sections from a triple negative breast tumor stained via immunohistochemistry for **(A)** H&E with hematoxylin staining nuclei dark blue-purple and eosin indicating cytoplasm in pink, **(B)** NODAL indicated by brown DAB staining, **(C)** TCRδ also stained brown with DAB, **(D)** Representative example of TCRδ (brown) found in a CAIX-positive region, stained pink with permanent red dye; scale bar = 40 μm. **(E)** Scoring for NODAL and CAIX expression in tumor sections in which γδ T cells were identified. Cases in which more than one slide was positive for TCRδ are indicated with −1, −2 designations. **(F)** Venn diagram depicting co-localization of γδ T cells (blue), NODAL (green), and CAIX (fuchsia). Percent overlaps are indicated.

Light microscopy and semi-quantitative scoring were performed by two pathologists. The entirety of each slide was assessed. Scores for CAIX were 0, absent; 1, weak and/or very focal staining; 2, strong but focal or moderate intensity; and 3, strong and extensive staining, as per our previous publication (22). The score reflects the intensity of staining observed in the majority of cells. NODAL was scored in the same manner on serial sections from the same cases. TCRδ staining was categorized as absent or present; when scored present, TCRδ+ cells were further identified as focal or diffuse. Only TCRδ+ cells within peri- and intratumoral stroma were considered. Co-localization between TCRδ+ cells and CAIX or TCRδ+ cells and NODAL was deemed positive or negative based on staining overlap. Proximity was defined as < 50 μm distance. Representative images were taken from a Nikon DS-U3 camera on Nikon eclipse 80i microscrope at 400 x (500 px bar = 40 μm). The Venn diagram in Figure 1F was created using a free online tool created by Dr. Tim Hulsen at http://www.biovenn.nl/venndiagram.tk/create.php, ^©^2003–2008.

### Primary γδ T Cells

Primary human γδ T cells were derived from healthy donor blood as described (10). In brief, peripheral blood mononuclear cells were isolated and cultured in media containing 1 μg/ml Concanavalin A and 10 ng/ml IL-2 and IL-4. T cells expanded together for 6–8 days, and then conventional αβTc were depleted by magnetic cell separation. For Vδ1 cultures used in migration assays, Vδ2 T cells were depleted from mixed T cells at the same time as αβTc [1 μl anti-TCRαβ PE (Biolegend) plus 0.5 μl anti-TCRVδ2 PE (Miltenyi Biotec) per million cells, followed by anti-PE beads, (Miltenyi Biotec)], and cells were supplemented with conditioned medium after depletion. Viability and fold expansion were routinely assessed *via* Trypan Blue exclusion and cell counting. When fed, cells were diluted to one million cells/ml with complete medium (RPMI 1640 with 10% FBS, heat-inactivated, 1 × MEM NEAA, 10 mM HEPES, 1 mM sodium pyruvate, 50 U/ml penicillin–streptomycin, and 2 mM L-glutamine—all from Invitrogen™, Thermo Fisher Scientific, Waltham, Massachusetts, USA) supplemented with 10 ng/ml IL-2 and IL-4. Subset composition and γδ T cell culture purities are provided in Table S1.

### Breast Cancer Cell Lines

Breast cancer target cell lines included MCF-7, T47D, and MDA-MB-231, all cultured in RPMI medium containing 10% FBS. MDA-MB-231 NODAL knockdown (shN) and scrambled control (shC) cell lines as well as T47D NODAL overexpresser (NOE) and empty vector (EV) control lines were established and characterized in our lab (33). They were cultured in RPMI containing 10% FBS and supplemented with 500 ng/ml Puromycin.

### *In vitro* Migration Assays (35)

For the experiment shown in **Figure 3A**, 60,000 MDA-MB-231 shN or shC cells in 600 μl complete medium were plated in the lower chamber of transwell plates (Corning #3421, 6.5 mm diameter inserts, 5.0 μm pore size, tissue culture treated) and allowed to adhere overnight. 20,000 αβ or γδ T cells in 100 μl serum free medium were plated in the top chamber and incubated for 3 h. Transwells were then washed in PBS, and then fixed in cold methanol for 15 min. After three washes in PBS, filters were carefully excised, placed on microscope slides (J. Melvin Freed, Frosted, Cat# 7,525 MF) and one drop DAPI mounting medium (Molecular Probes Prolong Gold antifade P36935) applied before placement of coverslips. Slides were stored at 4 degrees in the dark until visualization. 600 μl conditioned medium from MDA-MB-231 shN or shC cells was placed in lower chamber. Biological replicates shown in **Figure 3B** were done as follows: 50,000 Vδ1 γδ T cells in 100 μl serum free medium were plated in the top chamber and incubated for 3 h. Washing and fixing of membrane was done as described above.

Images were acquired on the Zeiss Axio Observer Z1 microscope such that all fields of view were stitched together to obtain an image of the entire transwell insert.

### Image Analysis

Images of migrated cells as identified by their DAPI-stained nuclei were analyzed using MetaXpress 6.0 software. Regions of interest (ROI) on 16-bit images were traced to encompass the entire filter; exemptions were drawn and subtracted to remove bubbles from the analysis. The value for net ROI, in pixels, was divided by one million. The net ROI was divided by one million to obtain a number below 100; this step is reflected in the 10^−6^ in the units. The Top Hat morphology filter (15–20 pixel diameter circle) was applied to remove artifacts. Nuclei with 10 −20 μm width displaying 10,000–15,000 intensity above background were considered to identify cells and were counted. True cells were defined with area ≤ 299 pixels. The total nuclei counted on the entire ROI from the insert were then divided by the ROI to achieve the # cells/pixel (x10^−6^) as depicted in the graphs. This was done to normalize the cell count to the area analyzed, to prevent skewing of results due to potential differences in excised filters or from loss of area due to bubbles.

### NODAL Stimulations

Unless otherwise stated, cells were stimulated with 100 ng/ml recombinant human NODAL protein (R&D Systems, catalog number 3218-ND/CF) for 4 h (cytotoxicity assays), 24 h or 4–10 days as indicated. Controls were NODAL vehicle control (NVC, 4 mM HCl in dH_2_O), 1.7 ng/ml carrier-free recombinant human TGF-β1 (BioLegend), 5 μg/ml anti-CD3 antibody (BioLegend, clone OKT3) or 200 μM pervanadate (4.1 μl 50 mM sodium orthovanadate, 1.2 μl 30% H_2_O_2_ plus 4.7 μl PBS per ml cell suspension).

### P19 Cell Stimulations

P19 mouse embryonal carcinoma cells were cultured and used periodically to verify the activity of recombinant human NODAL used in some assays. P19 cells were cultured in Alpha Minimum Essential Medium with ribonucleosides and deoxyribonucleosides, 7.5% bovine calf serum and 2.5% fetal bovine serum. P19 cells were seeded in 6 wells plate with 200,000 cells/well and grown in media with serum. The next day, media containing 10 μM SB431542 to suppress phosphoSMAD signals was added and incubated overnight. On the third day, the cells were washed with warm serum-free Alpha Minimum Essential medium and treated with rhNODAL 100 ng/mL (R&D system, cat#3218-ND/CF) for 1 hr at 37°C with 5% CO_2_ supplementation. After 1 h of treatment, cells were lysed and stored at −20°C for further western blotting analysis.

### Western Blotting

Cell lysates were prepared by adding M-PER Mammalian Protein Extraction Reagent containing Halt™ Protease and Phosphatase Inhibitor (both from Thermo Fisher Scientific) at 10 μl lysis buffer per million γδ T cells or 10 μl lysis buffer per 0.28 million target cells followed by 10 min incubation at room temperature. Cell lysates were then centrifuged for 15 min at 13,000 rpm at 4°C, after which supernatants were collected and 5 × reducing sample buffer [0.0625 M Tris/HCl pH6.8, 2% SDS, 20% glycerol, 0.05% β-mercaptoethanol, 0.025% (w/v) Bromophenol Blue] was added. Samples were boiled for 5 min and briefly centrifuged in a benchtop centrifuge before running on 10% SDS-PAGE gels. The mixed MW program on the Trans-Blot Turbo Transfer System (Bio-Rad, Mississauga, ON, Canada) was used to transfer proteins onto Immobilon-FL PVDF membranes (Millipore). Membranes were blocked for 40 min in 3% milk in TBST, followed by primary antibody incubation overnight at 4°C. Membranes were then washed and incubated with the corresponding species-specific HRP-labeled secondary antibody for 1 h, followed by further washing and finally detection using Clarity™ Western ECL Substrate (Bio-Rad). Primary antibody baths were prepared using PBS containing 2% bovine serum albumin and 0.05% sodium azide at the following dilutions: 1:3,000 mouse anti-human β-Actin (Santa Cruz, Danvers, MA, USA, clone C4); 1:2,000 rabbit anti-human β-Actin (Cell Signaling Technologies, Danvers, MA, USA); 1:2500 mouse anti-human NODAL (R&D Systems, clone 784410), 1:1000 anti-phospho-Smad2 (Cell Signaling, clone 138D4). Secondary antibodies were diluted in 3% milk in TBST (Tris buffered saline with Tween, 20mM Tris, 150 mM NaCl, 0.1% Tween 20) 1:10,000 goat anti-mouse IgG HRP (Bio-Rad); 1:20,000 goat anti-rabbit IgG HRP (Bio-Rad). The presence of multiple bands in some NODAL blots reflects different NODAL species corresponding to pro-NODAL, as well as processed NODAL (glycosylated/sialylated), and differ depending on cell type and conditions (33).

### Quantification of Bands on Western Blots

FIJI software (ImageJ Version 2.0.0-rc-15/1.49 m) was used to measure band intensities for phosphoSMAD2, Nodal and β-actin on 8-bit converted grayscale images using consistent rectangular regions of interest. Measured values for bands and background (region of same size beneath each band) were subtracted from 255, then net values for protein bands of interest and loading control bands (actin) were obtained by subtracting background values. Then, the ratios of the net protein bands to net loading control bands were calculated. Microsoft Excel version 15.3 (Microsoft, Redmond,WA, USA) was used for calculations.

### Flow Cytometry

#### γδ T Cell Immunophenotyping (9, 10, 38)

Live γδ T cells were gated on forward- and side-scatter properties and live/dead ZA staining. We used fluorescence minus one controls to set gates. Samples were acquired on BD FACS CantoII or Fortessa SORP X20 analyzers. Data were analyzed using FlowJo™ software version 10.6.0 for Mac (Becton Dickinson & Company, Ashland, OR, USA). In cases where Vδ1 + Vδ2 combined gates are indicated (**Figure 3A**, Figures S3A–C), the FlowJo tool “make or gate” under the Boolean dropdown menu was used to combine these gates.

#### Antibodies

For surface marker staining of γδ T cells, the following anti-human antibodies from BioLegend (unless otherwise indicated) were employed: TCRγδ PE (clone B1, 1:25); TCRγδ PE (Miltenyi, clone REA591, 1:10); TCRγδ BV421(clone B1, 1:10); TCR Vδ1 FITC (Miltenyi, clone REA173, 1:10); TCR Vδ2 PE (Miltenyi, clone 123R3, 1:100); TCR Vδ2 PerCP (clone B6, 1:25); CD27 AF700 (clone M-T271, 1:25); CD27 APC (clone M-T271, 1:25); CD45RA FITC (clone HI100, 1:25); CD69 AF700 (clone FN50, 1:4); CTLA-4 APC (clone L3D10, 5 μl); and PD-1 BV421 (clone EH12.2H7, 1:20).

For breast cancer cell line surface staining, anti-human MICA/B PE (clone 6D4, 0.1 μg); ULBP-2,5,6 (R&D systems, clone 165,903, 0.2 μg); ULBP-3 (R&D systems, clone 166,510, 0.04 μg); ULBP-4 (R&D systems, clone 709,116, 0.1 μg).

#### Surface Marker Staining

γδ T cells and breast cancer cell lines were re-suspended at 10 × 10^6^ cells/ml and stained with Zombie Aqua fixable viability dye in PBS (ZA, BioLegend) at a dilution of 1 μl/10^6^ cells for 15–30 min at room temperature in the dark. For γδ T cell staining, cells were stained directly with fluorochrome-conjugated antibodies diluted in FACS buffer [PBS containing 1% FBS and 2 mM EDTA (Invitrogen)] as indicated above. For the target breast cancer cell lines, cells were re-suspended at 10 × 10^6^ cells/ml and blocked with FACS buffer containing 50 μl/ml Trustain FcX (BioLegend) and incubated on ice for 30 min. Following blocking, cells were centrifuged and supernatants were removed such that 10 μl FACS buffer plus block remained. Antibodies and FACS buffer were added to 20 μl total volume, and cells incubated on ice 15–20 min followed by washing. Cells were then fixed in FACS buffer containing 2% paraformaldehyde (Sigma-Aldrich), stored at 4°C and acquired within 1 week.

#### Cell Trace Violet Proliferation Assay

γδ T cells were labeled as per the manufacturer's instructions with 1 μM Cell Trace Violet (Invitrogen), cultured for the indicated length of time, and were washed and re-suspended in FACS buffer prior to flow acquisition. Proliferation modeling was performed and statistics generated using FlowJo™ software, version 10.5.3.

#### Flow Cytometer Specifications

Cell samples were analyzed on a FACS CANTO II (Becton Dickinson, Mississauga ON) equipped with: an air-cooled 405-nm solid state diode, 30 mW fiber power output violet laser, with 450/50 and 510/50 band pass (BP) (502 long pass (LP) detector); a 488-nm solid state, 20-mW blue laser with 530/30 Bp (502 LP), 585/42 BP (556 LP), 670 LP (655 LP), and 780/60 BP (735 LP) filters; and a 633-nm HeNe, 17-mW red laser with 660/20 BP and 780/60 BO (735 LP) filters. Calibration was performed with CS&T beads (Becton Dickenson, Mississauga ON). Live singlets were gated based on forward and side-scatter properties and absence of fixable viability dye staining. Fluorescence minus one (FMO) controls were used to set gates. Analysis was performed using FlowJo™ software version 10.6.0.

### Fluorescence-Based Blocking/Cytotoxicity Assays (10)

#### Target Cell Labeling With Calcein AM

As per the manufacturer's instructions, target cells were labeled with 5 μM Calcein AM (CalAM, Invitrogen/Thermo Fisher Scientific). Cells were diluted to 30,000 cells/100 μl medium for cytotoxicity assays. For blocking assays, 4 μg blocking antibody (MICA/B, Biolegend, clone 6D4) was added to 400 μl cell suspension for each test in Eppendorf tubes, and from this, 100 μl/well was plated in a 96-well round-bottomed plate in triplicate and incubated at 37°C, 5% CO_2_ for 30 min. Mouse IgG (Sigma-Aldrich) was used as a control. Effector γδ T cells were re-suspended at a dilution of 6 × 10^6^ cells/ml in complete medium, then further diluted and added to target cells in 100 μl volumes to achieve the indicated effector:target (E:T) ratios; blocking assays were done at 20:1. Effectors and targets were incubated together at 37°C, 5% CO_2_ for 4 hr. Experimental controls were untreated and mouse IgG-treated cells (for the blocking assay). For CalAM fluorescence detection, plates were centrifuged and supernatants transferred to fresh 96-well plates (Costar, black plate, clear, flat bottom) and readings taken on a fluorimeter (FLUOstar Omega, BMG labtech). Controls were CalAM-labeled target cells incubated alone (spon = spontaneous release) and 0.05% Triton-X-100 (Thermo Fisher Scientific)-treated cells (max = maximum release). Percent lysis was calculated: [(test – spon)/(max – spon)] × 100%.

#### Flow Cytometric Cytotoxicity Assay (38)

Targets were labeled with 1 μM Cell Trace Violet 1 day prior to the assay. Targets were harvested and re-suspended in complete medium at 30,000/100 μl and plated 100 μl/well in a 96-well round-bottom plate. γδ T cells (effectors) were harvested and cell densities adjusted for each E:T ratio (1:1, 5:1,10:1, 20:1). Leftover γδ T cells were used for unstained, CTV only and Calcein AM Red Orange only staining controls. 100 μl effectors were added to targets and 100 μl/well media was added to target only wells; they were then incubated for 4 h at 37°C, 5% CO_2_. One Calcein AM Red Orange stock vial was reconstituted in 20 μl DMSO followed by further 1:5000 dilution in DMSO. Next, Calcein AM was diluted 1:100 in PBS. The 96-well plate containing effectors and targets was then centrifuged, pellets were re-suspended in 200 μl Calcein AM in PBS, and incubated at room temperature for 15 min in the dark. Finally, the plate was spun again, supernatants removed and pellets re-suspended in 200 μl FACs buffer [PBS containing 1% FBS and 2 mM EDTA (Invitrogen)]. Counting beads (Precision Count Beads^TM^, Biolegend, Catalog # 424,902) were diluted 1:4 in FACs buffer and transferred to FACS tubes (200 μl/sample) on ice to which 200 μl cell suspensions were added prior to acquisition on the Fortessa X-20.

#### Statistics

**Figure 2:**
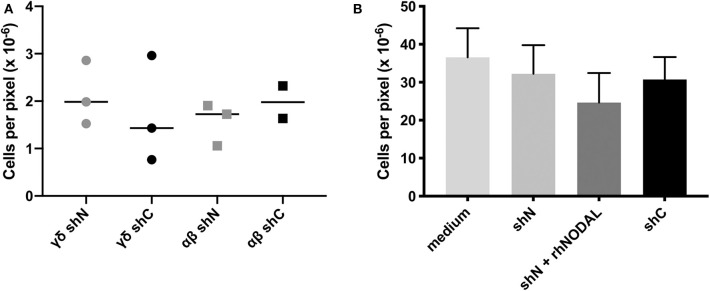
The NODAL-positive breast cancer cell secretome has no influence on γδ T cell migration. **(A)** No significant difference in migration of αβ and γδ T cells toward shN and shC plated in complete medium with 20,000 T cells in the top and 60,000 MDA-MB-231 cells in complete medium in the bottom chamber. Individual technical replicates are shown. **(B)** Compiled data from four independent Vδ1 migration assays toward medium or conditioned medium without or containing recombinant human NODAL (rhNODAL). Error bars are SEM.

Microsoft® Excel for Mac Version 15.30 was employed for paired 2-tailed Student's *t-*tests (Figure S3E). All other statistics were done using GraphPad Prism Version 8.2.1: Kruskal-Wallis and Dunn's multiple comparisons tests when Shapiro-Wilk normality tests failed because N was too small [(Figures S2A,C,D); one-way ANOVA analysis and Tukey's multiple comparisons (Figures 2A,B, Figure S2B)]; and two-way ANOVA with Bonferroni's pairwise multiple comparison *post-hoc* tests (**Figure 4**, Figure S4). The significance threshold was set at P < 0.05; asterisks indicate degrees of significance as indicated in the figure legends. Simple linear regression analyses were applied to data shown in **Figures 5C–E**, Figure S4P. The correlation matrix in **Figure 5F** shows calculated Pearson's correlation coefficients; the determined *P*-values were one-tailed.

## Results

### γδ T Cells Are Found in Areas in Which NODAL Is Expressed in Triple Negative Breast Tumors

Previously, we determined that γδ T cells are enriched in areas of hypoxia, as indicated by expression of carbonic anhydrase IX (CAIX), in estrogen receptor positive (ER+) breast tumors (22). We thus extended our studies to primary tumor tissues from a cohort of TNBC patients (Table 1) from which we stained serial sections of up to four different pieces of TNBC tumors from each patient (case). Representative examples are shown (Figures 1A–D). The hematoxylin and eosin (H&E) image depicts the invasive front of a triple negative breast carcinoma with large pleomorphic tumor cells showing intimate relationship to the stromal and immune microenvironment (Figure 1A). The tumor cells show strong cytoplasmic NODAL expression (Figure 1B). Scattered γδ T cells are seen in the vicinity of invasive tumor cell clusters (Figure 1C). Other examples from a different case are shown in Figures S1A–C. An image of γδ T cells in a CAIX-positive region from a third case are also shown (Figure 1D). We found γδ T cells in 45% (9/20) of cases studied on 13/39 slides. Scores for expression of NODAL and CAIX on these 13 slides are shown (Figure 1E).

In all cases in which both NODAL and γδ T cells could be detected, γδ T cells were found in close proximity to NODAL-expressing tumor cells; proximity was defined by a distance of < 50 μm. NODAL expression was observed in 78% of cases (7/9) and 85% of slides (11/13, Figure 1F). γδ T cells were found in regions of CAIX positivity in 100% of cases in which CAIX staining was evident (44%, 4/9 cases; 7/13 slides). Of seven slides from four patient tumors where CAIX and γδ T cell infiltration were both evident, in six (86%) they were co-localized, also with NODAL (46%, 6/13). It should be noted that γδ T cells were also found in areas in which neither NODAL nor CAIX were present. On all slides in which NODAL and CAIX were detected, regardless of γδ T cell infiltration, they were co-localized.

While this patient cohort is small and not powered enough to perform statistics, there appears to be no correlation of γδ T cell infiltration with patient age, invasive ductal carcinoma (IDC) size, grade, stage, or lymph node status (Table 1). Of the nine patients whose tumors contained γδ T cells, one had passed away as of February 2020 (11%); three of the eleven patients whose tumors lacked γδ T cells (27%) are deceased. Since NODAL is correlated with breast cancer progression, (36) and we found γδ T cells in close proximity to NODAL-expressing tumor cells, we decided to investigate the impact of NODAL on γδ T cells.

### NODAL Stimulation of γδ T Cells Does Not Alter Their Migration

Since chemotaxis is a major regulator of TME composition, we wanted to see whether NODAL had an influence on the migration of γδ T cells. In transwell assays, we tested migration of both αβ and γδ T cells toward MDA-MB-231 cells in which NODAL had been knocked down (γδ shN) compared to those in which NODAL was expressed (γδ shC) and observed no difference in the number of migrating cells (Figure 2A). A representative image of a transwell filter with migrated cells before, during and after processing for quantification is shown in Figures S2A–D, respectively. Since Vδ1 cells are often found within solid tumors (39) and investigators recently reported a majority of Vδ1 γδ T cell TIL in TNBC specifically, we enriched for Vδ1 γδ T cells and determined that they did not migrate preferentially toward conditioned medium from NODAL-expressing or NODAL knockdown cells. While the addition of recombinant human NODAL (rhNODAL) seemed to decrease migration somewhat, this difference was not significant. Compiled data from four independent migration assays with Vδ1 cells from four different donors are shown in Figure 2B. Results from the individual experiments in the compiled Figure 2B can be found in Figures S2E–H. Verification of NODAL expression in shN and shC cells used to produce conditioned medium for migration experiments can be found in Figures S2I,J.

### NODAL Stimulation Does Not Impact Activation Marker Expression, Proliferation or Maturation Profiles of γδ T Cells, but Longer Stimulation Time Results in Decreased Vδ2 TCR Expression

**Figure 3:**
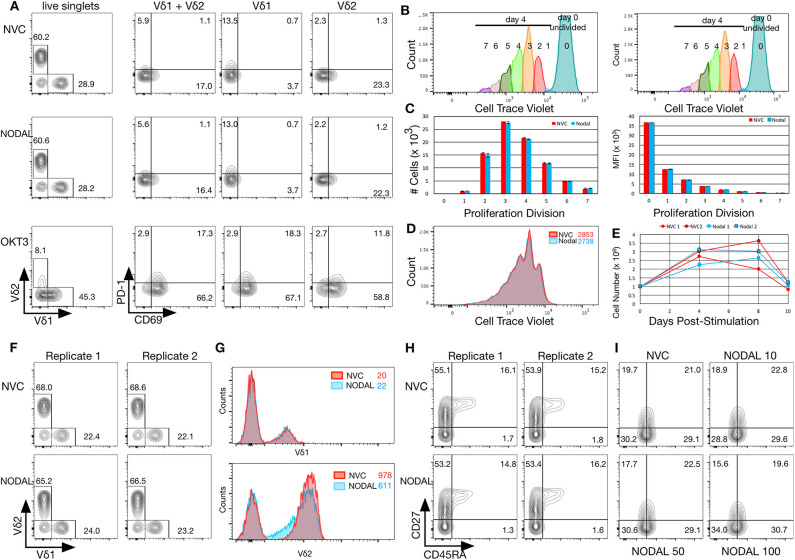
NODAL stimulation has no impact on activation markers, proliferation or maturation of γδ T cells but longer term exposure to NODAL results in decreased Vδ2 TCR surface expression. **(A)**, γδ T cells were subject to 24 h 100 ng/ml NODAL stimulation, and then surface expression of Vδ1 and Vδ2 TCR, PD-1, and CD69 were assessed *via* flow cytometry. NVC, NODAL vehicle control; anti-CD3 (OKT3) stimulation was done as a positive control for activation markers. This is a representative example of *n* = 3 independent experiments. **(B)**, Long term NODAL stimulation has no impact on γδ T cell proliferation. γδ T cells from the same culture as in A were labeled with Cell Trace Violet, then stimulated one time with NODAL vehicle control (NVC) or 100 ng/ml recombinant human NODAL. Flow cytometry was performed on days 0, 4, 8, and 10. Proliferation modeling analyses for the day 4 time point are shown. **(C)**, Cell numbers and median fluorescence intensities (MFI) of proliferation divisions for the experiment shown in B are shown; *n* = 2 technical replicates, error bars are SD. **(D)**, A representative example of histogram overlays from the day 4 time point of the experiment shown in B and C; MFIs are indicated. **(E)**, Cell counts on days 0, 4, 8, and 10 are plotted here for the two technical replicates done in the experiment shown in **(B–D)**. No significant differences were observed. **(F)**, Ten days after NODAL stimulation, cells cultured in parallel with the experiment in B-E were stained for Vδ1 and Vδ2 TCR expression; results from two technical replicates are shown. **(G)**, Histogram overlays and MFIs of TCR expression for representative examples from the experiment shown in **(F)**. **(H)**, For the experiment shown in **(F,G)**, maturation was assessed *via* CD45RA and CD27 staining; both technical replicates are shown. **(I)**, Maturation assessment after stimulating γδ T cells for 4 days with 10, 50, or 100 ng/ml recombinant human NODAL.

Compared to vehicle control, exposure to 100 ng/ml rhNODAL for 24 h had no impact on expression of Vδ1 or Vδ2 TCR, CD69, or PD-1 on the surface of primary human γδ T cells cultured for 14 days (Figure 3A). CTLA-4 was not detectible on these cells (data not shown). The Vδ1 + Vδ2 populations shown are a combination of those two individually gated cell types, combined using the FlowJo Boolean “make or gate.” Since the anti-Vδ2 TCR antibody outcompetes pan-γδ TCR antibody for binding, we do not show results for pan-γδ TCR staining (which would not include Vδ2 cells), but rather chose to combine Vδ1 and Vδ2 as indicated. Stimulation with OKT3, an anti-CD3 antibody, was included as a positive control for activation marker expression. As expected, both Vδ1 and Vδ2 TCRs were downregulated upon anti-CD3 stimulation; however, Vδ2 surface expression decreased more dramatically. Interestingly, Vδ1 appeared to have more basal PD-1 expression than Vδ2; in contrast, Vδ2 expressed more CD69 (Figure 3A, top panel, compare NVC Vδ1 and Vδ2 plots). Fluorescence minus one gating controls are shown in Figure S3A. These results were consistent with two other biological replicates done with γδ T cells from different donors; one other example is shown in Figures S3B,C.

To assess longer term impact of NODAL on γδ T cells, cells were labeled with Cell Trace Violet (CTV) and followed for 10 days. Samples were taken on days 0, 4, 8, and 10 for flow cytometric analysis. Proliferation modeling of data acquired on day 4 indicated no differences in proliferation between NVC and NODAL stimulated cells (Figures 3B–D), with proliferation indices averaging 1.81±0.01 and 1.815 ± 0.015, respectively. Cell counts for all of the time points for both technical replicates are shown in Figure 3E. Proliferation was measured via cell counting in a similar manner for three other cultures from two other donors (Figures S3D–F), with only one experiment showing some evidence of decreased proliferation of NODAL-treated cells 2 and 7 days post-stimulation but not at the end of culture (Figure S3D). In parallel, with the same γδ T cell culture used in Figures 3A–E, but left unlabeled, cells were stimulated with NODAL or NVC and then stained for flow cytometric assessment of Vδ1 and Vδ2 TCRs as well as maturation markers CD45RA and CD27. Proportions of Vδ1 and Vδ2 T cells were unaffected by NODAL stimulation (Figure 3F), and Vδ1 TCR expression levels remained unchanged (Figure 3G top panel); however, Vδ2 TCR expression levels decreased (Figure 3G bottom panel). This appears to occur as early as 4 days post-stimulation (Figure S3G). Maturation did not appear to be affected by long term NODAL stimulation (Figure 3H, FMOs in Figure S3I). Maturation was similarly unaffected in two other γδ T cell cultures subjected to a similar assessment (Figure 3I, Figure S3J), although perhaps there was a trend toward greater conversion of CD45RA^−^CD27^+^ central memory (CM) cells to CD45RA^−^CD27^−^ effector memory (EM) cells with higher NODAL doses after 4 days: at 10 ng/ml, CM/EM was 18.9/28.8 and at 100 ng/ml this was 15.6/34.0 (Figure 3I, FMOs in Figure S3K). An example of another 10-day NODAL stimulation is also shown, although it should be noted that cell viability for this culture by day 22 was no longer optimal and it appears that slightly more naïve cells were present in the NODAL-stimulated culture (Figure S3J).

### NODAL Expression Is Inversely Proportional to γδ T Cell Cytotoxicity

**Figure 4:**
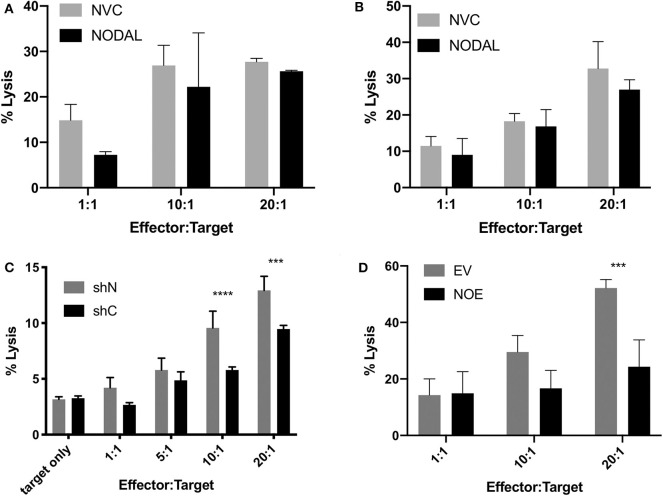
NODAL expression inversely correlates with susceptibility of breast cancer cells to γδ T cell cytotoxicity. **(A)**, NODAL stimulation during a 4-h Calcein AM-release cytotoxicity assay does not impact γδ T cell cytotoxicity against MCF-7 cells. NVC, NODAL vehicle control; *n* = 3 independent experiments. **(B)**, Long term NODAL stimulation has no impact on γδ T cell cytotoxicity against MCF-7 cells. γδ T cells were stimulated one time with NODAL vehicle control (NVC) or 100 ng/ml recombinant human NODAL, then 9 days later were co-incubated for 4 h with Calcein-AM labeled MCF-7 target cells at the indicated Effector:Target (E:T) ratio. **(C)**, Representative example in which day 21 γδ T cells and shC or shN MDA-MB-231 target lines were co-incubated for 4 h at the indicated E:T, and acquired *via* flow cytometry. ***P = 0.0002, ****P < 0.0001; *n* = 6 independent experiments. **(D)**, Overexpressing NODAL in T47D cells (NOE) confers significantly greater resistance to γδ T cell cytotoxicity as shown in a Calcein AM-release cytotoxicity assay; EV = empty vector control; A, B, D, Calcein AM assays; ***P = 0.0007; representative of *n* = 4 independent experiments. **(A–D)** Error bars are SD (3 technical replicates). *P*-values were calculated with 2 way ANOVA followed by Bonferroni multiple comparisons analysis.

Since NODAL is correlated with a poor prognosis in breast cancer, and prognosis is also associated with immune evasion, we chose to investigate whether NODAL is implicated in this resistance. Neither short- nor long-term stimulation of γδ T cells with exogenous recombinant human NODAL had any impact on γδ T cell cytotoxicity against MCF-7 breast cancer cells as shown in Calcein AM release assays (Figures 4A,B, Figures S4A–D). We then went on to investigate whether expression of NODAL in cancer cells could confer resistance, which constitutes a more physiologically relevant scenario, particularly since NODAL becomes upregulated under hypoxic conditions often found in tumors (32). For this, we made use of MDA-MB-231 NODAL knockdown and scrambled control cell lines as targets (35). Since these cell lines express GFP, which is not compatible with Calcein AM release assays, we turned to flow cytometric cytotoxicity assays to determine susceptibility of the lines to γδ T cell cytotoxicity. Indeed, we discovered that loss of NODAL confers susceptibility to γδ T cell killing, which is most significant at 10:1 and 20:1 effector:target ratios (Figure 4C, Figures S4E–I). We next utilized T47D cells [which have little endogenous NODAL expression (32)] transduced with an empty vector (EV) or a NODAL overexpression construct (NOE) as targets in our Calcein AM release cytotoxicity assays and found that NODAL overexpression confers resistance to γδ cell killing on T47D cells, again most prominently displayed at higher effector:target ratios of 10:1 and 20:1 (Figure 4D, Figures S4J–L). Verification of relative NODAL expression levels in these cell lines is depicted in Figure S4O. We plotted average percent lysis values for the 20:1 effector:target ratio for cytotoxicity assays shown in Figures 4C,D, Figures S4H,I,L,K—for which matched NODAL expression levels had been determined in Figure S4O—and performed linear regression analyses. The slope of the line of best fit was −21.33 and although the low *r*^2^ value of 0.3500 and position of data points outside the 95% confidence intervals indicated a poor fit, a significant negative association between % lysis and NODAL expression was nevertheless revealed (Figure S4P, *P* = 0.0427).

### Surface Expression of MICA/B Is Inversely Correlated to NODAL Expression

**Figure 5:**
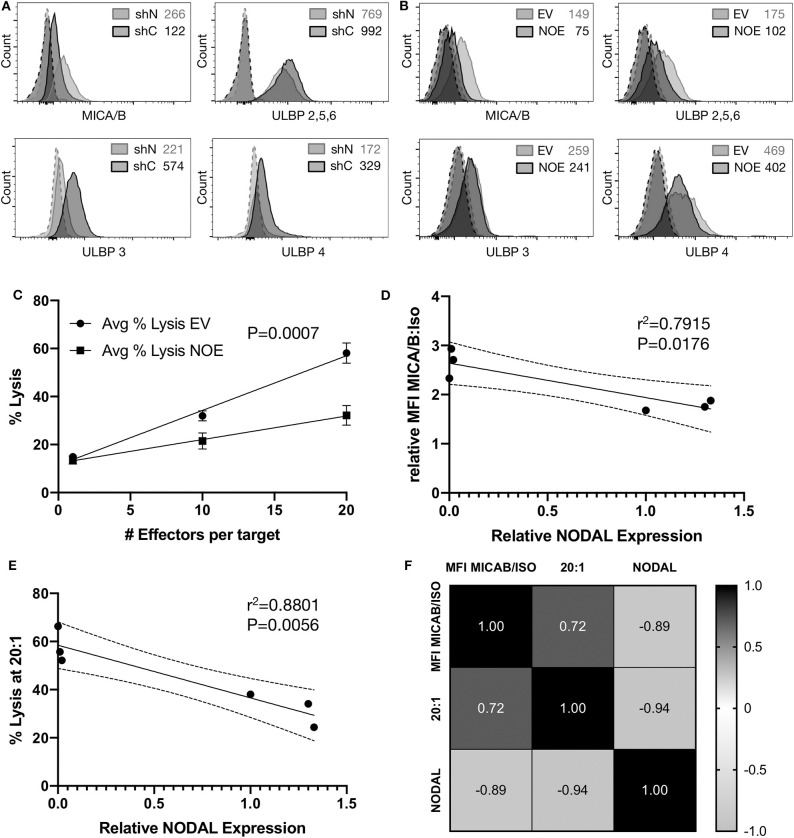
Surface expression of MICA/B is inversely correlated to NODAL expression. **(A)**, Representative examples of MICA/B, ULBP2/5/6, ULBP3, and ULBP4 tumor antigen surface expression on shC and shN MDA-MB-231 cells. Dashed lines are isotope controls. Median fluorescence intensity (MFI) values are indicated. **(B)**, Representative examples of MICA/B, ULBP2/5/6, ULBP3, and ULBP4 tumor surface antigen expression on T47D EV and NOE cells. Dashed lines are isotope controls. Median fluorescence intensity (MFI) values are indicated. **(C)**, Linear regression analysis on data from cytotoxicity assays shown in 4D, S4K,L. Avg = average. **(D)**, Relative ratios of MFI for MICA/B stained cells over isotype controls were plotted against relative NODAL expression as determined by densitometry on protein expression in lysates from matched cells shown in Figure S4O. **(E)**, Average percent lysis at 20:1 effector:target ratio from experiments shown in 4D, S4K,L were plotted against relative NODAL expression as determined by densitometry shown in Figure S4O. **(F)**, Correlation matrix showing Pearson's correlation coefficients from multiple variables analysis of percent lysis at 20:1, relative NODAL and MICA/B expression from experiments shown in Figure 4D, Figures S4K,L.

Flow cytometric assessment of the tumor surface antigens MICA/B, and UL-16 binding proteins (ULBP) 2–6 on shN and shC cells revealed that shN typically have higher surface MICA/B levels (Figure 5A, Figures S5A–C), but lower levels of all ULBPs tested compared to shC cells (Figure 5A). Similar analyses showed that the control EV line expressed higher levels of MICA/B (Figure 5B, Figures S5D–F), ULBP 2,5,6 and ULBP4 than NOE cells. Levels of ULBP3 were comparable on both lines (Figure 5B). As such, it appears that MICA/B surface expression and thus target cell susceptibility to γδ T cell cytotoxicity is inversely proportional to NODAL expression. We then blocked MICA/B on EV and NOE targets prior to cytotoxicity assays. Blocking EV with anti-MICA/B antibody reduced lysis down to a similar level to that of NOE targets (Figures S4M,N, compare EV MICA/B with NOE IgG), while blocking MICA/B on NOE targets had less impact than MICA/B blocking on EV (Figures S4M,N, compare IgG and MICA/B on EV and NOE). Linear regression analysis on averages from three independent cytotoxicity assays combined (Figure 4C, Figures S4K,L) indicated significantly decreased susceptibility to γδ T cell lysis of T47D NOE compared to EV targets (Figure 5C, *p* = 0.0007). After plotting relative MICA/B MFI over NODAL expression for the T47D EV and NOE cells used in Figure 5C, we performed simple linear regression. Narrowing this analysis to only T47D cells yielded a line of best fit with *r*^2^ = 0.7915 and a slope significantly different from 0 (Figure 5D, *P* = 0.0176). The same analysis of percent lysis at 20:1 vs. relative NODAL expression yielded a *P*-value of 0.0056 and *r*^2^ = 0.8801 (Figure 5E). Finally, analysis of percent lysis at 20:1, together with relative NODAL and MICA/B expression, yielded Pearson's correlation coefficients displayed in a matrix in which strong positive correlations were found between percent lysis and MICA/B expression (*r* = 0.72, *P* = 0.054) and negative correlations between percent lysis and NODAL expression (*r* = −0.94, *P* = 0.003) as well as NODAL and MICA/B expression (Figure 5F, *r* = −0.89, *P* = 0.009).

## Discussion

Alternative therapies for TNBC are in great demand (40) and the impact of the TME on γδ T cells is of great interest to those wishing to further develop γδ T cell immunotherapy (31). For example, altered tumor cell metabolism was addressed in a recent study describing harmful effects of LDL cholesterol on Vδ2 γδ T cell cytokine production and cytotoxicity against MDA-MB-231 *in vitro* and *in vivo*, which may well occur in the TME (41). We previously found that while hypoxia activates γδ T cells, at the same time low oxygen serves to downregulate surface expression and/or increase shedding of MICA by breast cancer cell lines leading to less efficient target cell recognition (22). Since NODAL is induced by hypoxia (32) and is correlated with breast cancer progression (42), we chose to investigate these particular elements of the TME and their influence on γδ T cell function.

We obtained formalin-fixed paraffin-embedded sections from twenty TNBC cases from which, in some cases, we could access slides from different parts of the same tumor. Gamma delta T cells were not equally distributed among these slides; in other words, the presence of γδ T cells in one section did not predict their presence in other tumor sections from another part of the same patient tumor, underlining the heterogeneity of tumors and infiltrating lymphocytes (8). As this was a relatively small cohort of patient tumors, we could not apply statistics to infer prognostic value of γδ TIL; however, there were enough examples of γδ T cell proximity to NODAL-expressing tumor cells to warrant further investigation of the potential impact of NODAL on γδ T cell function.

Hidalgo et al. (29) carefully assessed localization of γδ T cell TIL in a cohort of 26 TNBC tumors, comparing 14 IDC to 12 medullary breast cancer (MBC) cases. They found that most γδ T cells were in the tumor stroma in IDCs, but that in individual cases the cells could be found both in parenchyma and stroma (29). More recent pathological assessments of breast tumors no longer classify malignancies as MBCs, but rather TNBC cases are designated IDCs; as such, we cannot confirm Hidalgo's comparison. We can confirm, however, that γδ T cells could be found in close proximity to tumor cells, some of which expressed NODAL, but that most γδ T cells were localized in the adjacent tumor stroma. Since NODAL is a secreted protein, which would not be captured by IHC, it is reasonable to infer that the NODAL produced by tumor cells would come into contact with γδ T cells in the TME.

We looked for these effects by stimulating one time with exogenously administered rhNODAL and harvesting cells at various time points to determine functional outcome of this stimulus. While in many of our functional assays there appeared to be no significant influence of NODAL on γδ T cells, there were a few exceptions.

Although statistical analysis of compiled Vδ1 T cell migration assays did not yield significant differences, there was a trend toward lesser migration of these cells in the presence of rhNODAL (Figure 2B, Figures S2E–H) that may have become more clear had we altered incubation times for these assays. We also recognize that the presence of γδ T cells near NODAL-expressing tumor cells suggests that, if there is an inhibitory effect of NODAL on γδ T cell migration to the tumor, γδ T cells are able to overcome this, at least partially. Further exploration into chemokine receptor expression on γδ T cells after NODAL stimulation may be warranted. It should also be noted that the activity of rhNODAL was assessed periodically *via* P19 assays to ensure that the lack of response we observed in our assays was not due to lack of rhNODAL activity (Figure S6).

Vδ2 cells expressed CD69 after anti-CD3 stimulation, which has been found by others to indicate activation in the form of degranulation and production of proinflammatory cytokines (43), whereas Vδ1 cells upregulated PD-1. Such subset-specific responses to anti-CD3 stimulation are reminiscent of the work of Kress et al. (44) who stimulated Vδ1 and Vδ2 cells with PMA/Ionomycin or LPS and measured resulting gene expression changes in the two subsets, which were considerable, with ~50% being subset specific. Unfortunately, access to the complete gene lists is no longer available online; as such, we were unable to determine whether CD69 or PD-1 were assessed in their analyses. While no noticeable TCR downregulation had occurred at 24 h post-NODAL stimulation (Figure 3A), 4 days later, Vδ2 TCR internalization was evident (Figure S3H), which was also seen in other experiments at 10 days post-stimulation (Figure 3G, Figure S3I). Typically, the TCR is internalized upon TCR stimulation, as seen in Figure 3A after exposure to anti-CD3, and so this decreased receptor expression indicates some form of activation that we have, as of yet, been unable to pinpoint. Vδ1 TCR expression remained unchanged. Such differential responses of Vδ1 and Vδ2 subsets to stimuli, which we can assess with our polyclonal γδ T cell cultures, may prove useful in the development of subset-specific γδ T cell immunotherapies. One limitation of our study was our use of activated expanding primary γδ T cells, which may have masked subtle effects of NODAL stimulation. In future studies, “untouched” γδ T cells could be used in stimulation assays and also extended to additional readouts such as cytokine release and CD107a degranulation assays.

Most γδ T cell immunotherapy development currently focusses on Vδ2 cells, yet Vδ1 cells are often found in solid tumors. In a very early study utilizing frozen sections from five breast carcinomas, Bank et al. (19) found both Vδ1 and Vδ2 γδ T cell TIL, with slightly higher prevalence of Vδ2 cells, but their cohort was small. In contrast, Peng et al. (45) generated tumor-derived TILs from breast, prostate and melanoma tumors, finding greater numbers of Vδ1 than Vδ2 γδ T cell TIL derived from the epithelial malignancies (breast and prostate), but not in cultures derived from melanoma. While the authors went on to show immunosuppressive qualities of Vδ1 TIL-derived γδ T cells, these assays were conducted only after expansion of cells in high levels of IL-2; considering the inherent plasticity of γδ T cells (46), these immunosuppressive effects may well have been induced by culture conditions and may not reflect the activity of the cells *in situ*.

In contrast, the activity of γδ T cell TIL in TNBC *in situ* has been painstakingly investigated in a recently published study in which γδ T cells were identified in frozen TNBC tumor sections from nine patients, isolated by laser capture microdissection and subjected to single cell sequencing analysis. These analyses confirmed a polyclonal population of γδ T cells had infiltrated TNBC tumors and that these expressed CD69 and the pro-inflammatory cytokines IFNγ and TNFα; only a minor fraction (<20%) expressed IL-17 (30). Since different combinations of TCRγ and TCRδ chains confer distinct antigen recognition capabilities (30), if the response of γδ T cells to NODAL stimulation is TCR dependent, effects on individual clones would have been lost in our current analyses. As such, a study of the impact of NODAL on clonal populations may be of interest, or single cell RNAseq (39) could be employed to tease out individual responses. This was beyond the scope of our current study, but could be considered moving forward.

While NODAL belongs to the TGF-β family, we did not observe the effects reported by Peters et al. (17) with respect to enhancement of γδ T cell cytotoxic activity. In contrast, we found no impact on cytotoxic activity upon addition of rhNODAL to our cytotoxicity assays (Figure 4A) or with longer-term γδ T cell stimulation prior to co-culture with targets (Figure 4B), although considering the shift from CM to EM observed after 4 days of NODAL stimulation (Figure 3B), this may have been evident had we assessed cytotoxicity after 4 days instead of 10 days, since by 10 days NODAL stimulation there was no difference in maturation status of γδ T cells compared to control NVC-stimulated cells (Figure 3I).

We found that the ability of target cells to produce NODAL decreases their susceptibility to γδ T cell killing (Figures 4C,D, Figures S4E–N,P). Previous work from our laboratory documented variable endogenous NODAL levels across breast cancer cell lines, and that MDA-MB-231 cells express more NODAL than T47D (42), which we have confirmed (Figure S4O). Furthermore, the MDA-MB-231 shN NODAL knockdown cells produce more NODAL than T47D EV cells (Figure S4O compare lanes 1, 3 and 5 with relative intensities for shN of 0.6, 0.3, and 0.2 to lanes 8, 10, 12, and 14 for EV, all 0). Linear regression analysis of percent lysis from cytotoxicity experiments performed with six different donor cultures vs. NODAL expression in 231 shN/shC and T47D EV/NOE targets revealed a significant negative correlation between NODAL expression and susceptibility to γδ T cell killing (Figure S4P). The data points are more closely clustered when applied only to T47D EV/NOE (Figure 5E), yet the slopes of the lines from these two analyses are nearly the same (−21.33 and −21.9).

There is a significant inverse correlation of NODAL with MICA/B on the tumor cell surface (Figures 5A,B,D,F). Blocking EV with anti-MICA/B antibody reduced lysis down to a similar level to that of NOE targets, suggesting that this is indeed an important mechanism by which γδ T cells recognize and target T47D breast cancer cells (Figures S4M,N); however, the greater resistance of MDA-MB-231 compared to T47D cannot be solely attributed to NODAL, and we unfortunately did not measure matched MICA/B expression levels for shN and shC targets concurrent with our cytotoxicity assays. MICA shedding played a significant role in the evasion of breast cancer cell lines to γδ T cell killing under hypoxia in our previous study (22). Altogether, our work confirms the findings of Aggarwal et al. (18) who showed that susceptibility of breast cancer cells to killing by Vδ2 γδ T cells was dependent on MICA/B surface levels. Considering that our assays were performed with primary γδ T cells expanded from many different donors (Table S1), which is expected to confer a great deal of inter-donor variability, we observed a remarkable negative correlation between the lysis of T47D targets and their expression of NODAL (Figures 5E,F). A very strong negative correlation between MICA/B and NODAL expression was also evident (Figures 5D,F). Thus, NODAL perhaps mediates tumor cell escape by somehow regulating expression of surface MICA, the exact mechanism of which remains to be determined. The interaction of NODAL with γδ T cells in the TME may well comprise another example of the tissue sensing adaptate function of γδ T cells (15), the understanding of which deserves further attention to optimize their clinical potential.

## Data Availability Statement

All datasets presented in this study are included in the article/Supplementary Material.

## Ethics Statement

The studies involving human participants were reviewed and approved by Research Ethics Guidelines, Health Research Ethics Board of Alberta—Cancer Committee. The patients/participants provided their written informed consent to participate in this study.

## Author Contributions

GS and L-MP contributed to research design. GS and ID conducted experiments. Data analysis was carried out by GS, ID, EK, JH, and MK. GS wrote the manuscript. All authors provided feedback and approved the final version.

## Conflict of Interest

The authors declare that the research was conducted in the absence of any commercial or financial relationships that could be construed as a potential conflict of interest.

## Abbreviations

αβTc: αβ T cells
bp: band pass
BrCa: breast cancer
CAIX: carbonic anhydrase IX
CalAM: Calcein AM
CTV: Cell Trace Violet
ER: estrogen receptor
E:T: effector:target ratio
FACS: fluorescence acquired cell sorting
FBS: fetal bovine serum
FMO: fluorescence minus one
γδ T cells: gamma delta T cells
H&E: hematoxylin and eosin
HRP: horseradish peroxidase
IDC: invasive ductal carcinoma
IHC: immunohistochemistry
IL: interleukin
lp: long pass
mAb: monoclonal antibody
MACS: magnetic cell sorting
MBC: medullary breast cancer
MICA: MHC class I polypeptide-related sequence A
MFI: median fluorescence intensity
NKG2D: natural killer group 2, member D
PBMCs: peripheral blood mononuclear cells
PBS: phosphate buffered saline
PIC: protease and phosphatase inhibitor cocktail
rhNODAL: recombinant human NODAL
shC: scrambled control
shN: NODAL knockdown BrCa
spon: spontaneous release
TBST: tris-buffered saline plus 0.05% Tween-20
TCR: T cell antigen receptor
TGFβ: transforming growth factor β
TIL: tumor infiltrating lymphocytes
TME: tumor microenvironment
TNBC: triple negative breast cancer
ZA: Zombie Aqua fixable viability dye
ZNIR: Zombie Near Infrared fixable viability dye.
