# Supplementary material for: Aberrantly Expressed Embryonic Protein NODAL Alters Breast Cancer Cell Susceptibility to γδ T Cell Cytotoxicity
Source: Front Immunol. 2020 Jun 19;11:1287. doi: 10.3389/fimmu.2020.01287 (PMC7319087; doi:10.3389/fimmu.2020.01287)
Supplement: Supplementary file 1 [file Table_1.docx]

| ID |  | Day | % Vδ1 | % Vδ2 | % γδTCR+Vδ1-Vδ2- | % Purity | Figure(s) |
| --- | --- | --- | --- | --- | --- | --- | --- |
| ED3 | **1A** | 20 | n.d. | n.d. | n.d. | 96.1 | 2A |
| DM3 | **2A** | 14 | 73.3 | 0.3 | 21.7 | 95.3 | 2B, S2E |
| BH2 | **3A** | 14 | 73.1 | 0.1 | 24.8 | 98.0 | 2B, S2F |
| BH11 | **3B** | 20 | 11.8 | 83.2 | 4.1 | 99.1 | 5C, E, F, S4L, M |
| LL4 | **4A** | 14 | 70.6 | 0.3 | 22.3 | 93.2 | 2B, S2G |
| JM5 | **5A** | 14 | 68.5 | 0.1 | 20.6 | 89.2 | 2B, S2H |
| CV3 | **6A** | 13 | 28.9 | 60.2 | 8.7 | 97.8 | 3A, S3A |
| CV3 | **6A** | 21 | 22.4 | 68.0 | 9.4 | 99.8 | 3B-H, S3I |
| CV2 | **6B** | 21 | 3.4 | 92.1 | 3.4 | 98.9 | 5C, E, F, S3D, S4K |
| KV4 | **7A** | 17 | 5.6 | 82.2 | 11.8 | 99.6 | 3I, S3G, S3K |
| NP1 | **8A** | 22 | 2.9 | 84.4 | 1.0 | 88.3 | 4A, B, S3F, S3H, S3J |
| SM5 | **9A** | 21 | 46.0 | 42.2 | 7.4 | 95.6 | 4C, 5C, E, F, S4N |
| XX2 | **10A** | 21 | 8.6 | 71.1 | 3.9 | 83.5 | 4D, S3E, S4A, S4C |
| CM7 | **11A** | 13 | 2.5 | 93.8 | 1.6 | 97.9 | S3B,C |
| CM6 | **11B** | 21 | 3.5 | 92.2 | 2.8 | 98.5 | S4I |
| FE1 | **12A** | 23 | 9.8 | 66.0 | 9.8 | 85.6 | S4B, S4D |
| MJ1 | **13A** | 15 | n.d. | n.d. | n.d. | 97.5 | S4E |
| SF1 | **14A** | 20 | 8.3 | 82.5 | 5.8 | 96.6 | S4F, G |
| KB1 | **15A** | 20 | 3.9 | 92.4 | 2.1 | 98.4 | S4H |
| NG4 | **16A** | 15 | 17.4 | 63.6 | 4.5 | 85.5 | S4J |

**Table S1** Subset percentages and purities of donor-derived γδ T cell cultures. On the indicated day cells were harvested, stained with fixable viability dye followed by the following antibodies: pan γδ TCR, Vδ1 TCR and Vδ2 TCR and acquired by flow cytometry. The purity is calculated as the sum of %Vδ1, %Vδ2 and %γδTCR+Vδ1-Vδ2-. Their use in experiments for figures in this manuscript are listed. n.d. = no available data
