# Supplementary figures and images for: Aberrantly Expressed Embryonic Protein NODAL Alters Breast Cancer Cell Susceptibility to γδ T Cell Cytotoxicity
Source: Front Immunol. 2020 Jun 19;11:1287. doi: 10.3389/fimmu.2020.01287 (PMC7319087; doi:10.3389/fimmu.2020.01287)

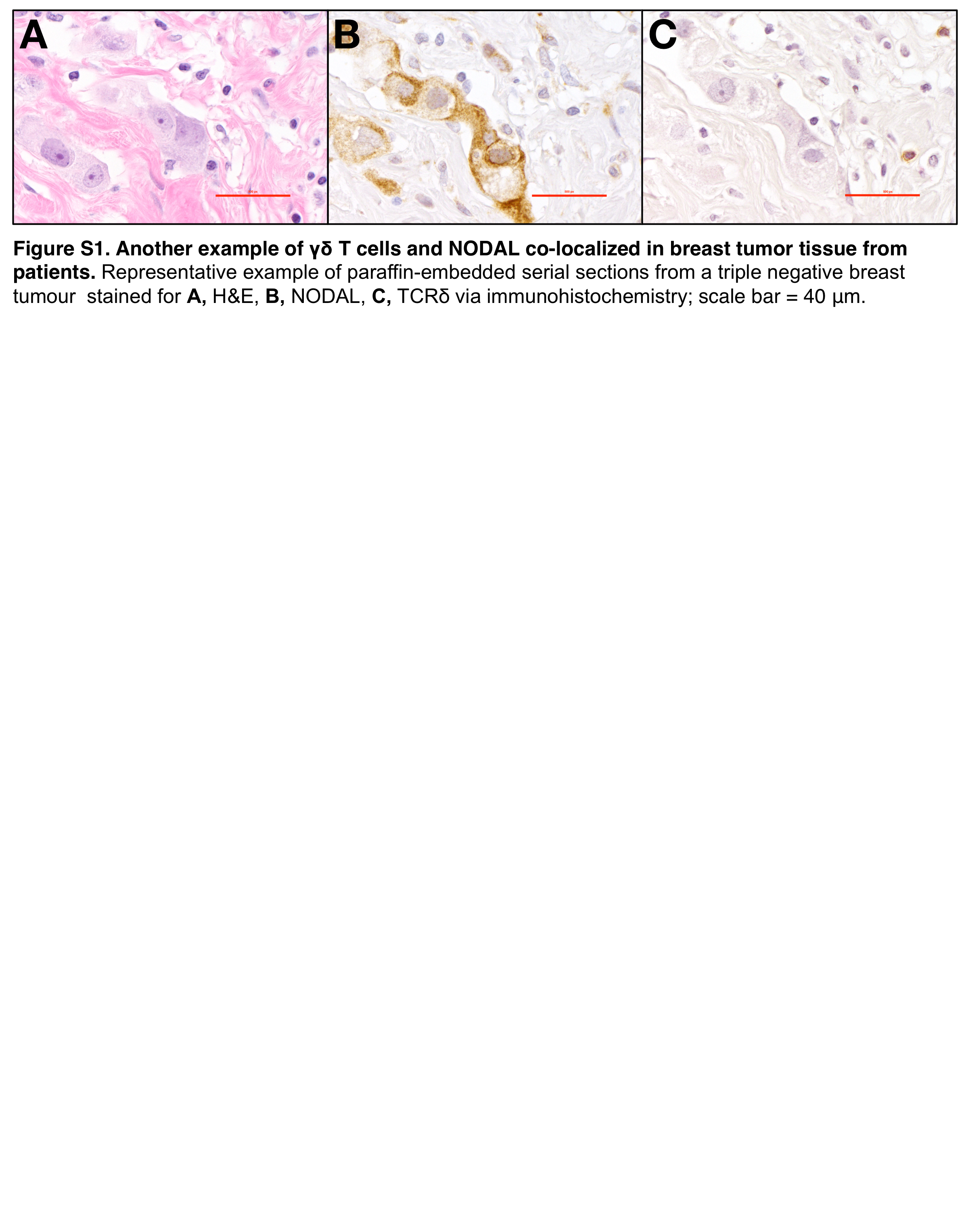

Supplement: Supplementary file 2 [file Data_Sheet_1.zip › Figure S1.TIFF]

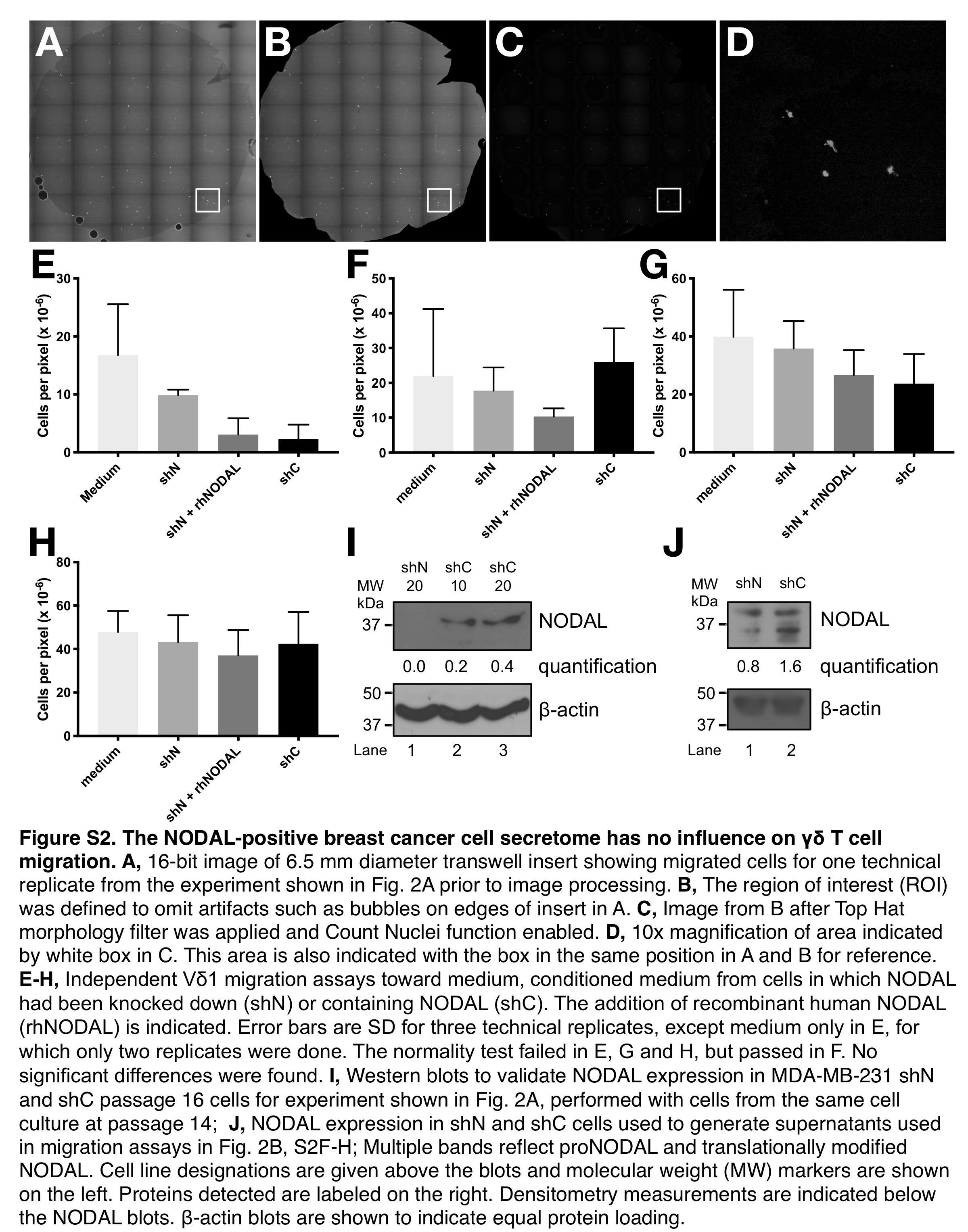

Supplement: Supplementary file 2 [file Data_Sheet_1.zip › Figure S2.TIFF]

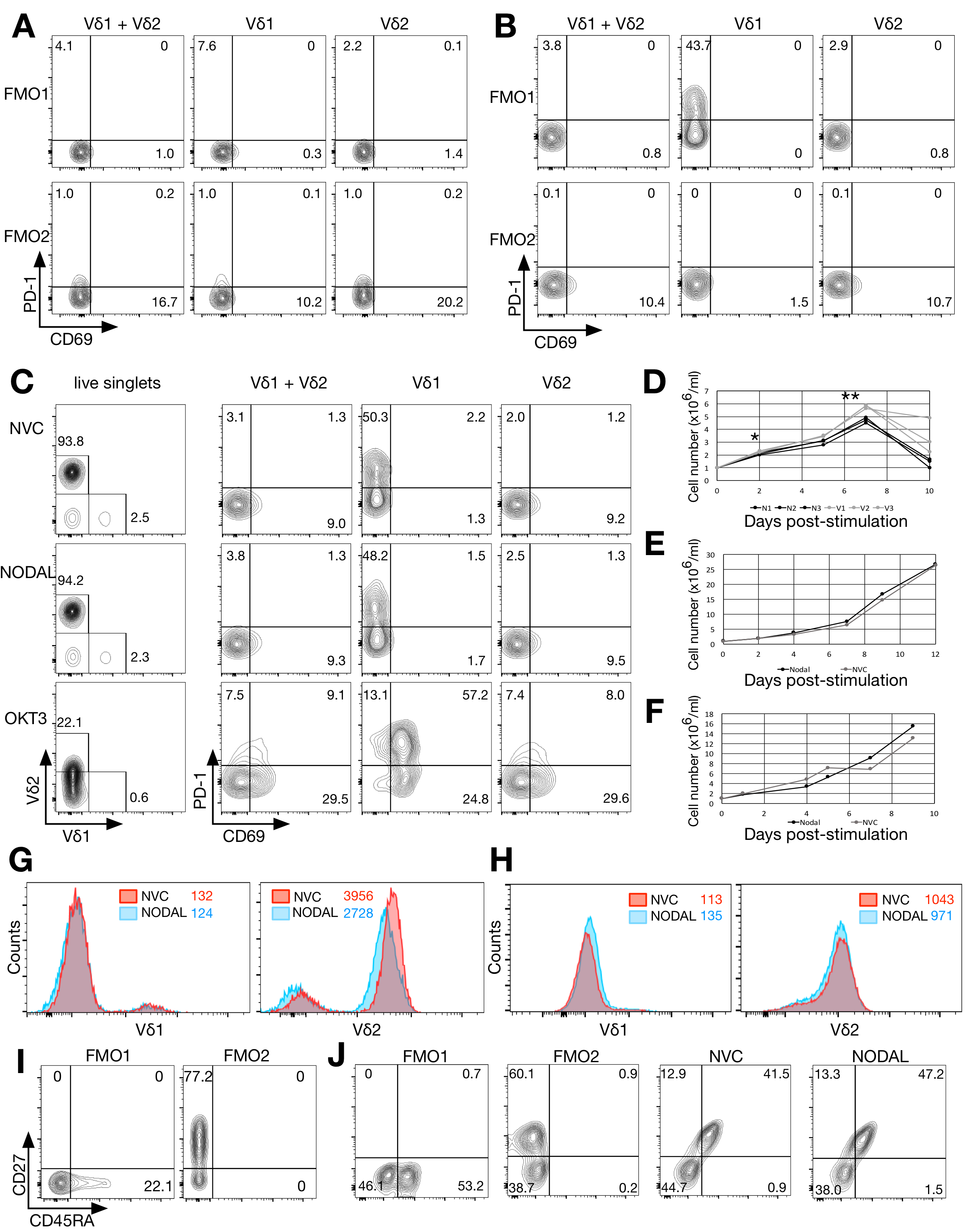

Supplement: Supplementary file 2 [file Data_Sheet_1.zip › Figure S3 - 1.TIFF]

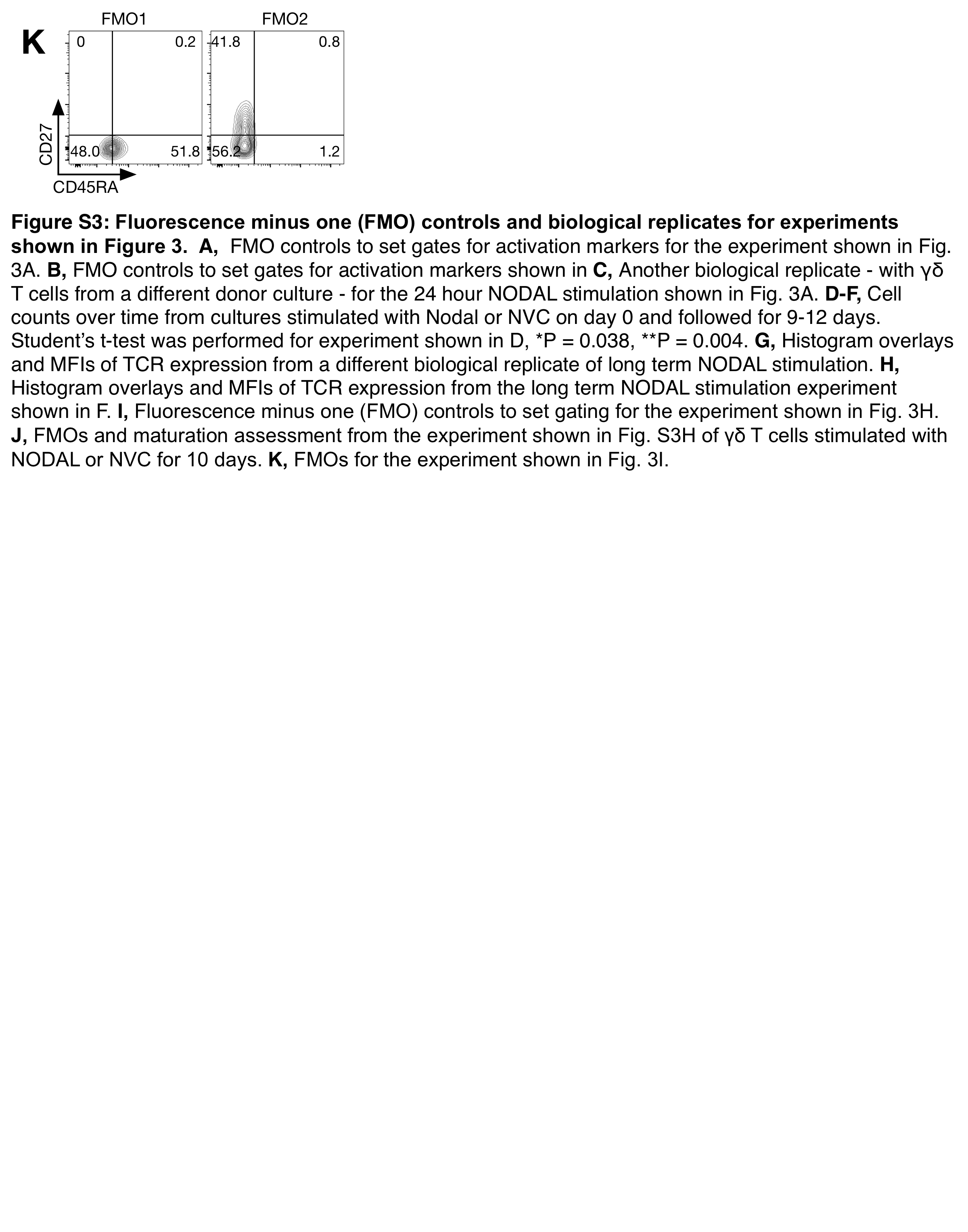

Supplement: Supplementary file 2 [file Data_Sheet_1.zip › Figure S3 - 2.TIFF]

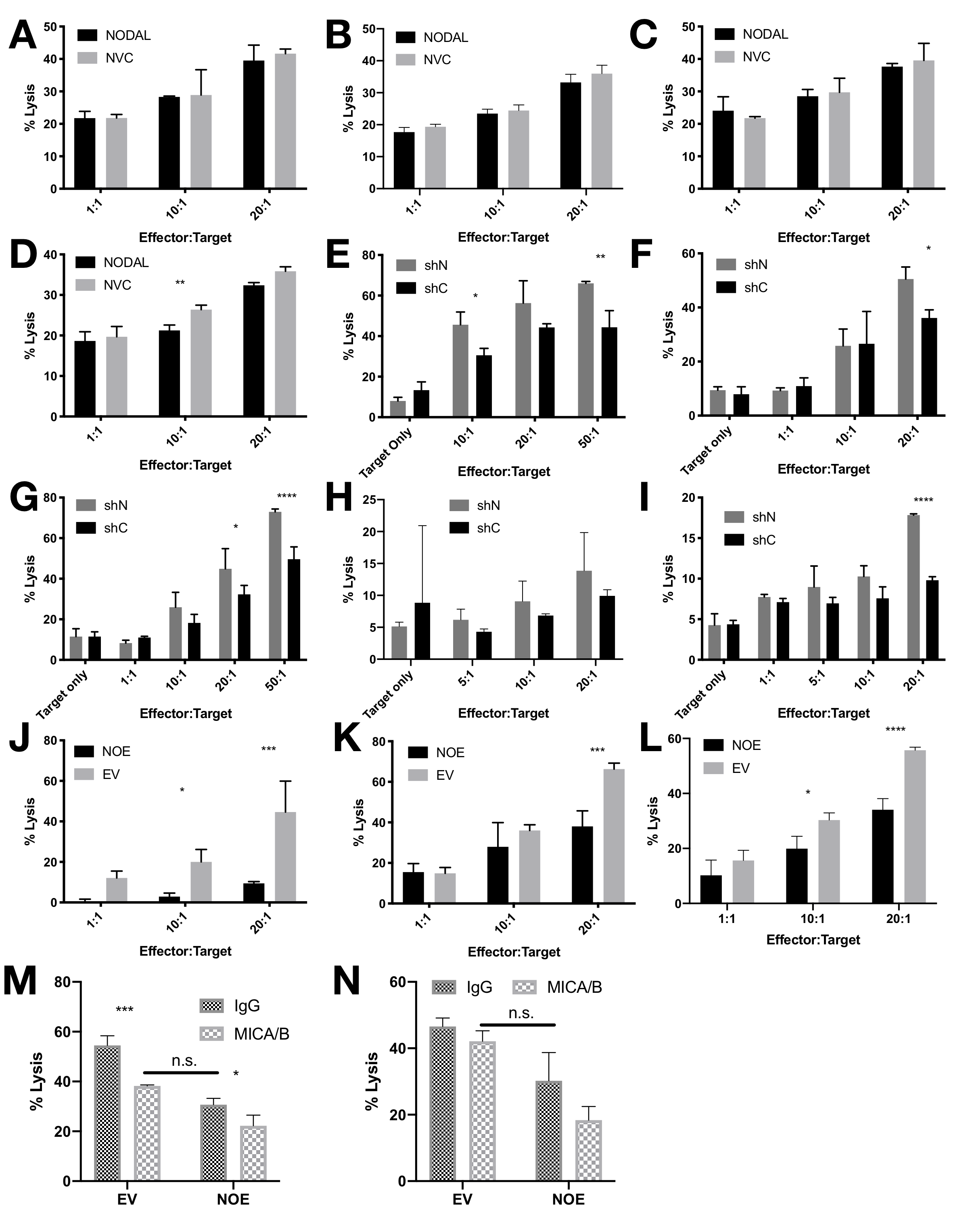

Supplement: Supplementary file 2 [file Data_Sheet_1.zip › Figure S4 - 1.TIFF]

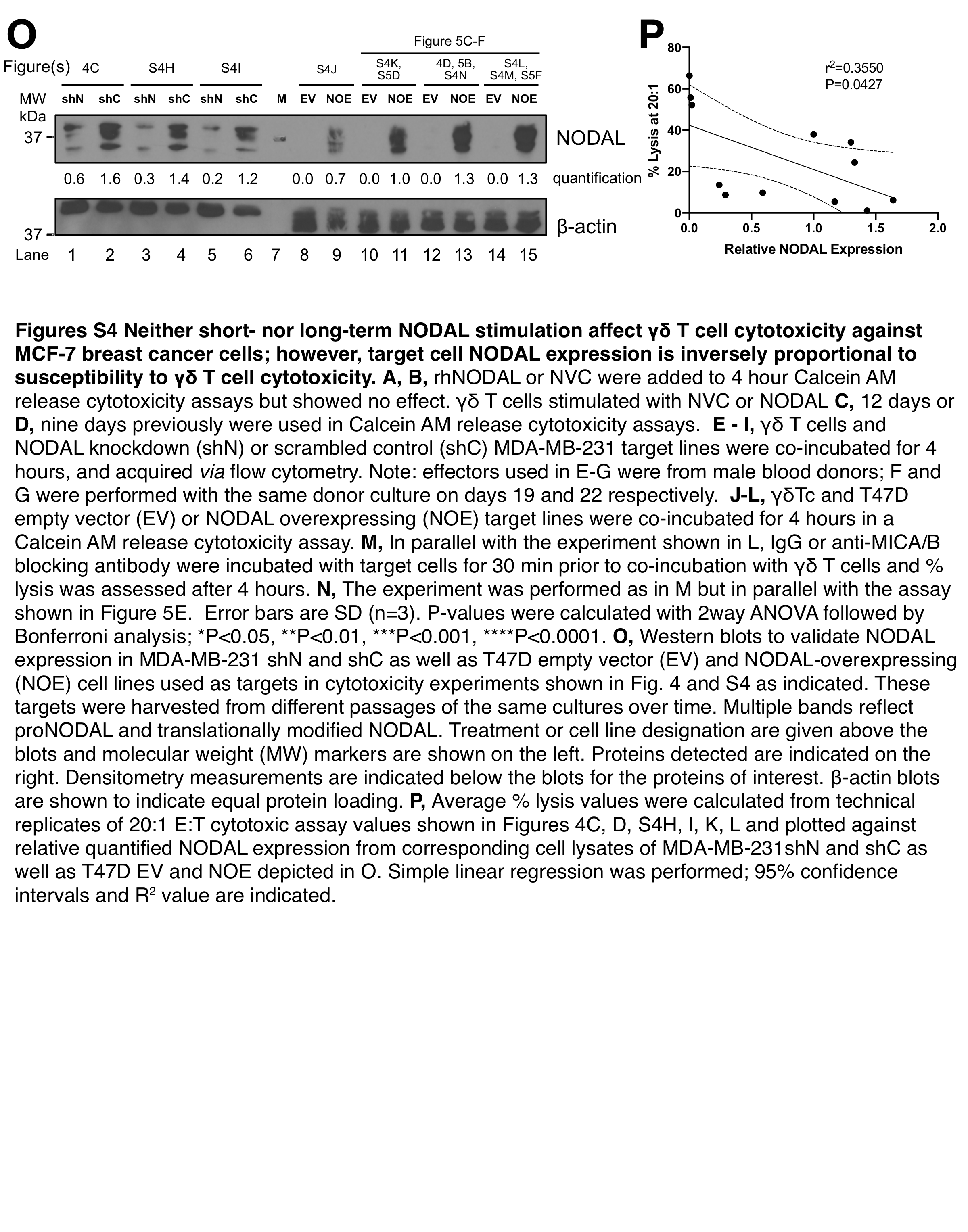

Supplement: Supplementary file 2 [file Data_Sheet_1.zip › Figure S4 - 2.TIFF]

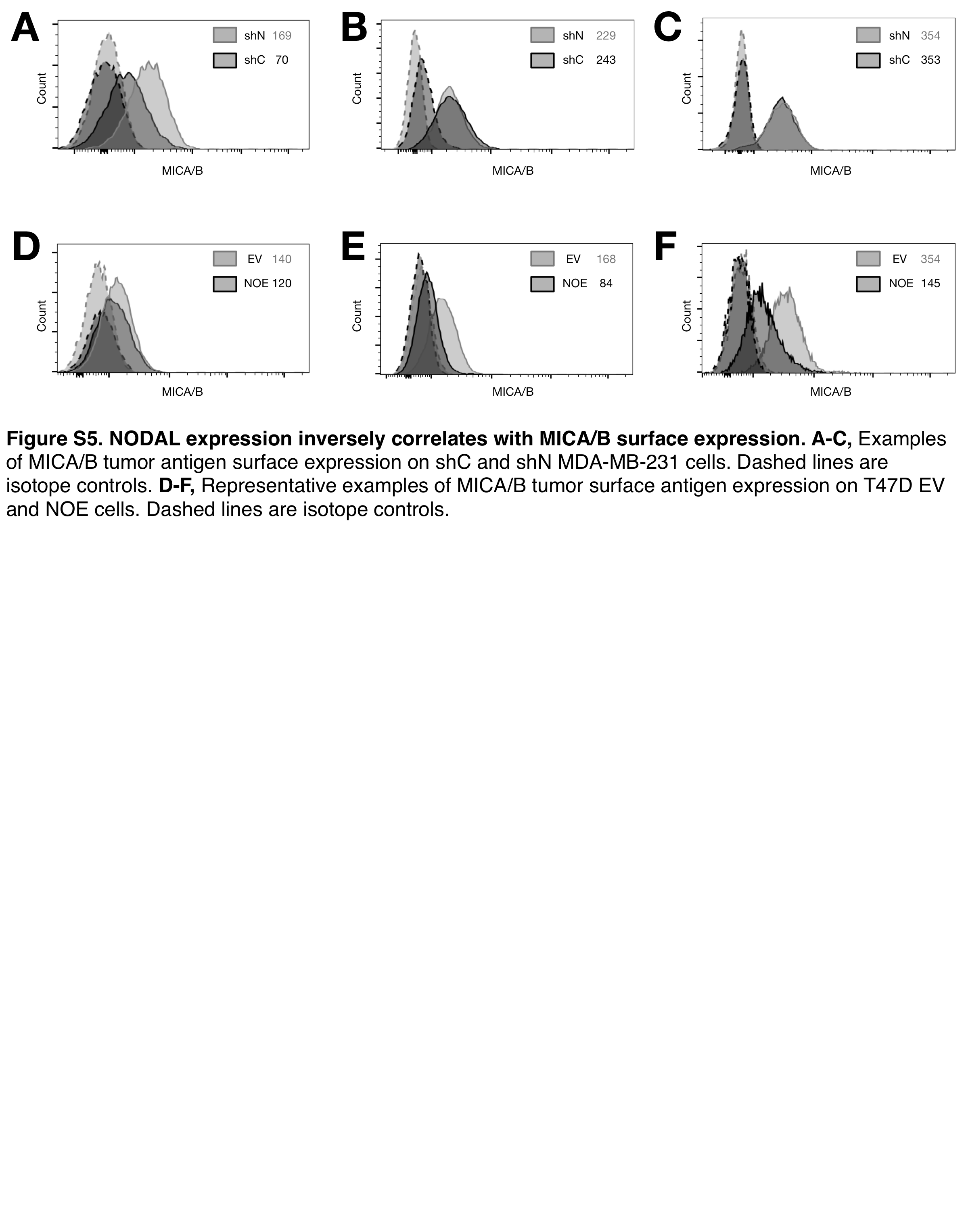

Supplement: Supplementary file 2 [file Data_Sheet_1.zip › Figure S5.TIFF]

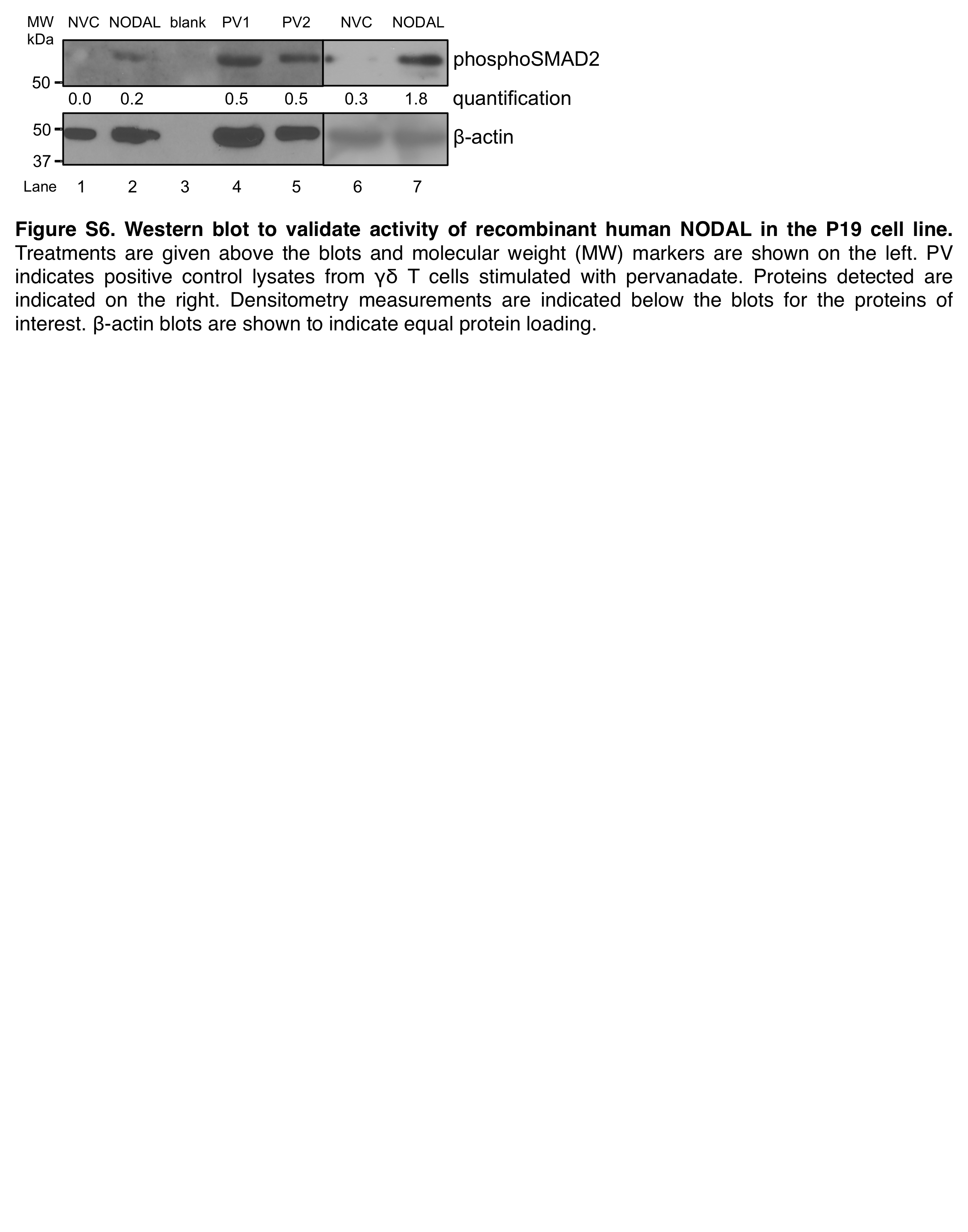

Supplement: Supplementary file 2 [file Data_Sheet_1.zip › Figure S6.TIFF]
